# eHealth literacy and attitudes towards use of artificial intelligence among university students in the United Arab Emirates, a cross-sectional study

**DOI:** 10.3389/fdgth.2025.1574263

**Published:** 2025-09-04

**Authors:** Zufishan Alam, Aminu S. Abdullahi, Shamma Nayea Salem Alnuaimi, Hanouf Abubaker Al Shaka, Saif Slayem Saif Alderei, Ahmed Abdulla Ali Alhemeiri, Hayma Khorzom, Hamad Jumaa Mubarak Almaskari, Khalid Abdulrahman Almaamari, Khalifa Al seiari, Mohammed Al Saadi, Nasser Al Shamsi, Omar Al zaabi, Saoud Altamimi, Azhar T. Rahma

**Affiliations:** ^1^ School of Health Sciences, Hamdan Bin Mohammed Smart University, Dubai, United Arab Emirates; ^2^ Institute of Public Health, College of Medicine and Health Sciences, United Arab Emirates University, Al-Ain, United Arab Emirates

**Keywords:** artificial intelligence, chatGPT, eHealth literacy, healthcare, higher education, United Arab Emirates

## Abstract

**Introduction:**

With the rapid digitalization of healthcare information and the increasing dependability on online health resources, it has become crucial to understand digital health literacy and the use of emerging AI technologies like ChatGPT among stakeholders. This is of particular importance in the United Arab Emirates which has the highest internet penetration rates.

**Method:**

This study aimed to assess eHealth literacy and the factors influencing it among university students in the United Arab Emirates. Their attitudes towards ChatGPT use were also explored. Data from participants, studying in the public universities of UAE, was collected between April–July 2024 using eHEALS and TAME Chat GPT instruments.

**Results:**

Results indicated a mean eHealth literacy score of 29.3 out of 40, with higher scores among females and those in health–related disciplines. It was also found that students with higher eHealth literacy perceived ChatGPT as more useful in healthcare, despite their concerns about its risks and potential to replace healthcare professionals.

**Discussion:**

The findings from the study underscore the need of development of tailored digital health curricula, to enhance eHealth literacy particularly in subgroups showing lower literacy scores. Moreover, it is also imperative to develop guidelines for responsible and ethical AI use in health information seeking.

## 1 Introduction

The constant advancement of new technologies nowadays has transformed every possible aspect of our lives, including healthcare. This shift is not merely a trend; rather an established paradigm that requires active adaptation. The concept of health literacy was proposed for the first time in 1970s (1). Although an evolving concept, it is broadly defined as the degree of the ability to find, understand, and appraise health information and services in order to make health-related decisions (2). Health literacy is a complex multidimensional term, encompassing four dimensions, including ability to (1) retrieve pertinent health information, (2) comprehend the health information accessed, (3) analyse and infer the health information obtained, and (4) utilize the gathered health information to make a decision to enhance health outcomes (3).While health literacy remains fundamental to public health, the digital revolution has introduced the concept of eHealth literacy (4). eHealth literacy is defined as the ability to evaluate health information from digital sources and use this acquired knowledge to address or resolve health-related problems (5).

Both traditional health literacy and eHealth literacy aim to enable individuals to comprehend and apply health information. However, they differ significantly in their approaches. While conventional health literacy relies on healthcare professionals, including doctors, dentists, public health workers, and health volunteers, to deliver health information, digital health highly depends on the availability and accessibility of the internet and involves obtaining information from electronic sources such as Internet websites, social media platforms, and artificial intelligence (AI) tools (6, 7). According to Norman and Skinner, eHealth literacy comprises six different literacy components: computer literacy, health literacy, information literacy, scientific literacy, media literacy, and traditional literacy (5).

Given that the most recent estimation of Internet users worldwide is 5.44 billion, constituting nearly 67.1% of the world's population, the importance of eHealth literacy cannot be overlooked in today's digital era. Asia accounts for the largest number of online users, with over 2.93 billion people. Saudi Arabia and the United Arab Emirates has the highest internet penetration rate in the region, reaching 99% of their population (8).

Digital health has emerged as a rapidly expanding field of research, with numerous studies conducted on assessment of eHealth literacy and various aspects of its utilization among individuals over the past decade globally. A study assessing eHealth literacy levels among nursing students in 2014 in South Korea indicated that 51.1% participants had high eHealth literacy rates. 70% of the students found the Internet very useful for making health-related decisions (9). Yet, only a few students felt confident in differentiating between high- and low-quality health resources online. Another European study that focused on assessing eHealth literacy in medical students in 2020 concluded that 53.2% of the participants perceived their eHealth skills as poor or very poor with a significant proportion feeling inadequately prepared for digital health skills (10). Another study in Austria in 2022 identified that while students recognized the importance of eHealth, a substantial portion, lacked the understanding of digital health technologies, and declared the use of the Internet as a tool for communication rather than for health education (11).

Various validated scales, including but not limited to, eHealth Literacy Scale (eHEALS) have also been used in literature to measure eHealth literacy levels. For instance a study conducted in Greece demonstrated a mean score of 31.9 out of 40 on the eHEALS among the participants, indicating moderate levels. Medicine and dentistry students were found to score the highest (mean score of 33.7), while students from other health sciences had the lowest (mean score of 29.8), with no significant difference in scores based on the academic level (12). Another Australian study highlighted that higher the use of digital engagement and communication among students, higher is its reflection on the students' eHealth literacy scores, indicating better knowledge (13).

Within the MENA region, the Persian eHealth Literacy Scale (eHEALS) has been used in an Iranian study to measure health literacy of university students. Results suggested low overall literacy level, with factors such as department, education level, health status, monthly income, gender and preferred health-related information websites significantly associated with scores (14). Another Iranian study concluded that the percentage of students using the Internet to search for information on different aspects such as disease symptoms, physical illnesses, existing treatments, and diagnoses were 70%, 67.1%, 65%, and 63.1% respectively (15). In Jordan, research reported student difficulty in differentiating between high- and low-quality resources (16). Narrowing it down to the Gulf Cooperation Council (GCC) countries, a study in the United Arab Emirates (UAE) found that for 74.7% of participating Emirati adolescents and young adults, health-related decisions were based on information obtained from social media platforms, whereas 83.7% considered health authorities to be the most trustworthy source (17). However, the assessment of eHealth literacy of students in the UAE still remains unexplored, that this study aims to address.

With the introduction and use of artificial intelligence, various language learning models (LLMs) have been widely employed in higher education. Of the used LLMs, ChatGPT has gained extensive popularity among the students (18). It has been extensively used to access, understand and use online health information, thus serving as digital health information tool. Research has outlined its applications and potential limitations in healthcare education, research and practice (19). With the accelerating interest and use among students, work has also been done to assess students' attitudes towards ChatGPT use. Recently a study validated an instrument to evaluate attitudes and usage of ChatGPT among students, using a large multinational sample from five Arab countries (19). The instrument called “Technology Acceptance Model Edited to Assess ChatGPT Adoption” (TAME-ChatGPT) was found to be an adequate, useful, reliable and valid tool to assess adoption of ChatGPT among university students as well as healthcare students (20, 21).

Despite that the UAE has the highest penetration rate and considerably high percentage of the population adolescents rely on Internet resources to obtain their health information, as well as use Chat GPT (17, 22), there is limited research on assessment of eHealth literacy among the university students. This study aimed to assess the overall level of eHealth literacy as well as the influencing factors among the university students in the UAE. It also aimed to examine the relationship between students' attitudes towards Chat GPT use and their eHealth literacy levels.

## 2 Methods

This study followed a cross-sectional method with survey used as the primary tool for data collection.

### 2.1 Study setting, population, and sampling

The population in this study consisted of university students currently enrolled in public or private universities across the seven emirates of the United Arab Emirates. University students were selected as the target population because they are expected to employ these literacy skills in their daily lives, especially as they transition to independent healthcare decision-making and engage in self-directed exploration of health-related literature. The sample included students aged 18 years and above, irrespective of gender and nationality. Students pursuing higher-level studies, in addition to the Bachelors, such as Diplomas, Masters and Doctoral degrees were also included in the study. The minimum sample size was estimated to reach a confidence interval of 95% with a margin of error of 5%. Given that the total population of university students exceeded 100,000, and no prior studies testing health literacy among this population in the UAE existed at the time, the response distribution was set at 50%. The sample size was calculated using Rao soft, resulting in a recommended sample size of 380 which was adjusted to 471 to account for non-response (24%).

To reduce bias and ensure that all university students had an equal chance of participating, all students meeting the inclusion criteria from both public and private universities in the seven emirates were invited. Representation from different universities was ensured to provide a comprehensive understanding of the target population. To reach this diverse population, a multi-channel recruitment strategy was utilised including official invitation emails sent to the administrative offices of all licensed universities. Student engagement was further boosted through annual undergraduate and postgraduate research conferences, where the study was promoted. Additional outreach occurred through direct campus engagement, university WhatsApp groups, email forwarding by faculty, and snowball sampling to expand coverage.

### 2.2 Data collection

A nationwide cross-sectional internet-based survey encompassed undergraduate and postgraduate students from various universities in the United Arab Emirates. All governmental and private universities were invited to participate in the study to maximize student participation. The questionnaire was disseminated to the students via their official academic emails and facilitated using SurveyMonkey, chosen for its accessibility and convenience among the student population, as well as built-in features that enables seamless organization of the data for subsequent analysis. Each participant was required to submit only one response, and responses were kept anonymous to ensure confidentiality. Students were sent weekly reminders to ensure participation. The questionnaire's language level was set to be accessible to high-school students, ensuring that participants would not experience difficulty in responding.

To ensure a comprehensive and diverse sample, the survey was distributed using a combination of methods. Direct interactions with students were conducted on university campuses, allowing for face-to-face engagement. Additionally, the survey was shared through digital channels such as WhatsApp and email, enabling participation from students who were not physically present on campus. To further broaden the participant pool, a snowball sampling strategy was also implemented. Initial respondents were encouraged to distribute the survey to other university students within their social circles, thereby extending the survey's reach beyond the initial group.

Ethical approval for the study was received from the Social Sciences Ethics Committee Institutional Review board of the University (ERSC_2024_4426). Approval from the research and ethics committees of each institution was additionally obtained. Consent to participate in the study was gathered through the online survey platform. Participants were asked to give their informed consent before beginning the survey. It was also made clear that participants could withdraw from the study at any point. To protect respondents' privacy, no personally identifiable information was collected. Data collection was carried out over approximately four months, from April 1, 2024, to July 1, 2024.

### 2.3 Evaluation tool

The eHEALS questionnaire was used as the evaluation tool. eHEALS is an 8-item measure designed to assess participants’ knowledge and perceived skills in finding, evaluating, and applying electronic health information (5). The eHealth literacy score was measured on a 5-point scale ranging from 1 (strongly disagree) to 5 (strongly agree). Scores ranged from 8 to 40, with higher scores indicating increased perceived skills in utilizing electronic knowledge to assess and implement health-related decisions. A validated Arabic version of the questionnaire was also distributed, which was previously used by Bergman et al. (23) to assess eHealth literacy among Arab-speaking residents in Sweden (23). To assess attitudes towards ChatGPT usage, validated Arabic version of TAME ChatGPT was used (20). The tool consists of three constructs, each with respective subscales. These included the usage construct with subscales on perceived usefulness, behavioural factors, perceived risk of use and perceived ease of use; the attitude construct with subscales on perceived risk, anxiety and attitude to technology; and the health application construct with two subscales on perceived healthcare utility and perceived potential to replace healthcare providers respectively (21).

### 2.4 Statistical data analysis

Categorical variables were summarized using frequencies and percentages, while quantitative variables were summarized using mean and standard deviation. The dependent variable, eHealth literacy, was categorized, based on eHEALS scores. For both the eHealth literacy scale and the TAME-ChatGPT scale, scores were assigned to the responses to each item/question as thus: Strongly agree = 5, agree = 4, neutral = 3, disagree = 2, and strongly disagree = 1. For eHealth literacy, the total score for each respondent was computed as the sum of the individual scores of the scale items. The median score was then computed and used to categorize the score into high (above the median) or low (equal or below the median). Scores between 8 and 26 indicated limited eHealth literacy, while scores between 27 and 40 indicated sufficient eHealth literacy (23). For TAME-ChatGPT, the overall and subscale scores were computed as the total score for each item, and these were then divided by the number of items to obtain the average scores. Hence, each computed average score, whether overall or subscale, had a highest possible score of 5 and a lowest possible score of 1.

Associations between eHealth literacy level (categorical) and other categorical variables were assessed using the Chi-squared test; while associations between eHealth literacy level (categorical) and TAME-ChatGPT scores (quantitative) were assessed using the Independent *T*-test. Binary logistic regression analysis was used to explore factors independently associated with eHealth literacy level. Five predictors, including age, gender, college, PubMed as source of literature, and grade point average (GPA), were initially chosen *a priori* based on the literature and the investigators' judgment. Univariate models were fitted with each predictor, and those with a *p*-value of less than 0.25 were included in the multivariable model. All predictors met this threshold except GPA, which was subsequently dropped from the multivariable model. Multicollinearity among the predictors was assessed using variance inflation factor, with a threshold of 2. All the adjusted VIFs were below 1.1, suggesting a lack of multicollinearity. Model fit was assessed using the Pearson Chi-square goodness-of-fit test.

All inferential statistics were performed at 5% level of significance. Data were analyzed using R (version 4.4.1).

## 3 Results

### 3.1 Participants' demographic characteristics

A total of 237 students participated in the survey with a median age of 21 years (Table 1). The majority were females (59%), Emiratis (78%), from public universities (75%), had a bachelor's degree (83%), and were in health-related disciplines (54%).

**Table 1:** Demographics of participants (*N* = 237) taking part in the survey.

Characteristic	Frequency (%)
Age, mean (SD)	21.6 (4.7)
Age category	
≤20	95 (40%)
>20	141 (60%)
Gender	
Female	141 (59%)
Male	96 (41%)
Nationality	
Emirati	186 (78%)
Expat	51 (22%)
Education	
Diploma degree	19 (8.0%)
Bachelor's degree	196 (83%)
Postgraduate	22 (9.3%)
University type	
Public	177 (75%)
Private	60 (25%)
College	
Medicine and health sciences	129 (54%)
Science & technology	61 (26%)
Others	47 (20%)
Study year	
1st year	48 (20%)
2nd year	42 (18%)
3rd year	59 (25%)
4th year	66 (28%)
5th year and above	22 (9.3%)
GPA, mean (SD)	3.3 (0.48)
GPA category	
<3.3	88 (41%)
≥3.3	128 (59%)

### 3.2 Ehealth knowledge and practice

Overall, the participants had a mean eHealth literacy score of 29.3 (SD = 3.6) out of possible 40 translating to 75%. Most of the participants reported they knew “how to find helpful health resources on the internet” (84%), “what health resources are available on the internet” (78%), “how to use the Internet to answer my questions about health” (86%), “where to find helpful health resources on the Internet” (82%), and “how to use the health information they found on the internet to help themselves” (80%) (Table 2). The majority also agreed that they had the skills they needed to evaluate the health resources they found on the Internet (70%), that they could differentiate high-quality health resources from low-quality ones on the internet (72%), and that they felt confident using information from the internet to make health decisions (56%). A detailed summary of these responses can be found in Table 3. Both instruments, the Arabic eHEALS and TAME-ChatGPT, showed good internal consistency, with Cronbach's alphas of 0.87 and 0.72, respectively.

**Table 2:** eHealth literacy score by selected participants’ characteristics and sources of information.

Characteristic	High *n* (%)	Low *n* (%)	*p*-value
Overall	81 (34.2%)	156 (65.8%)	**—**
Age category			0.140
≤20	27 (28%)	68 (72%)	
>20	53 (38%)	88 (62%)	
Gender			0.057
Female	55 (39%)	86 (61%)	
Male	26 (27%)	70 (73%)	
College			0.026*
Medicine and Health Sciences	53 (41%)	76 (59%)	
Sci. & Tech.	13 (21%)	48 (79%)	
Others	15 (32%)	32 (68%)	
University type			0.900
Public	61 (34%)	116 (66%)	
Private	20 (33%)	40 (67%)	
GPA			0.700
<3.3	31 (35%)	57 (65%)	
≥3.3	42 (33%)	86 (67%)	
PubMed/Scholar as a source of information			0.018*
No	53 (30%)	124 (70%)	
Yes	28 (47%)	32 (53%)	
Mayo/Cleveland as a source of information			0.900
No	67 (34%)	128 (66%)	
Yes	14 (33%)	28 (67%)	
UpToDate/Web medic/NMedics as a source of information			0.900
No	68 (34%)	132 (66%)	
Yes	13 (35%)	24 (65%)	

* Statistically signficant.

**Table 3:** Participants’ responses to the eHealth literacy scale.

Constructs on the eHEALS Scale	*N* (%)
I know how to find helpful health resources on the Internet	
Strongly Agree	64 (27%)
Agree	134 (57%)
Disagree	12 (5.1%)
Strongly Disagree	10 (4.2%)
Undecided	17 (7.2%)
I know what health resources are available on the internet	
Strongly Agree	55 (23%)
Agree	130 (55%)
Disagree	13 (5.5%)
Strongly Disagree	6 (2.5%)
Undecided	33 (14%)
I know how to use the Internet to answer my questions about health	
Strongly Agree	86 (36%)
Agree	117 (49%)
Disagree	13 (5.5%)
Strongly Disagree	6 (2.5%)
Undecided	15 (6.3%)
I know where to find helpful health resources on the Internet	
Strongly Agree	74 (31%)
Agree	121 (51%)
Disagree	16 (6.8%)
Strongly Disagree	4 (1.7%)
Undecided	22 (9.3%)
I know how to use the health information I find on the Internet to help me	
Strongly Agree	66 (28%)
Agree	124 (52%)
Disagree	7 (3.0%)
Strongly Disagree	6 (2.5%)
Undecided	34 (14%)
I have the skills I need to evaluate the health resources I find on the Internet	
Strongly Agree	61 (26%)
Agree	106 (45%)
Disagree	18 (7.6%)
Strongly Disagree	7 (3.0%)
Undecided	45 (19%)
I can tell high-quality health resources from low-quality health resources on the Internet	
Strongly Agree	71 (30%)
Agree	99 (42%)
Disagree	13 (5.5%)
Strongly Disagree	11 (4.6%)
Undecided	43 (18%)
I feel confident in using information from the Internet to make health decisions	
Strongly Agree	43 (18%)
Agree	89 (38%)
Disagree	31 (13%)
Strongly Disagree	16 (6.8%)
Undecided	58 (24%)

### 3.3 Sources of eHealth information

**Figure 1:**
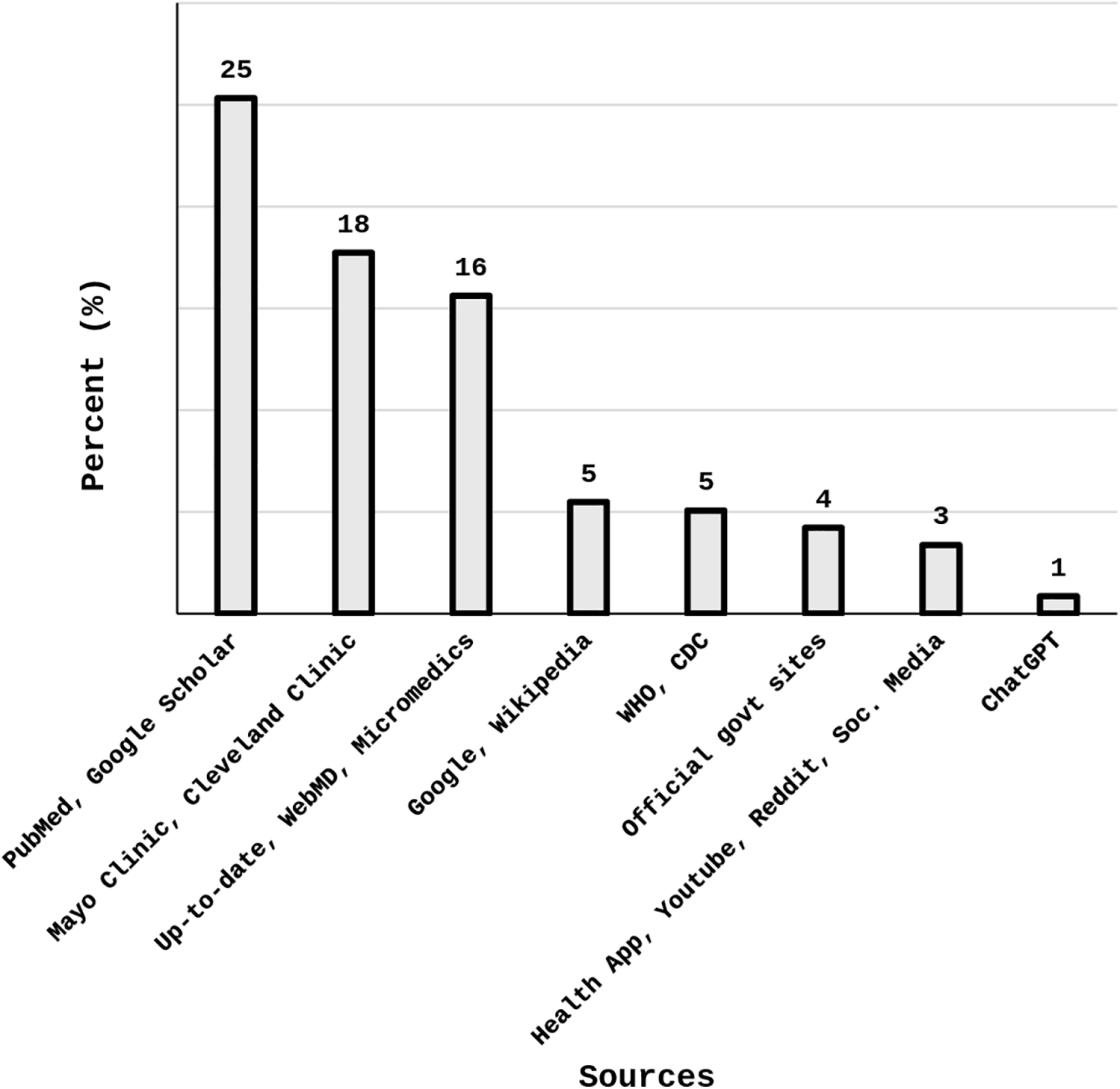
Participants’ sources of information on the internet.

Internet sources used by the participants are summarized in Figure 1. The most common sources of information on the internet were PubMed and Google Scholar (25%); followed by Mayo Clinic and Cleveland Clinic (18%); and Up-to-date, WebMD and Micromedics (16%); Google and Wikipedia (5%); WHO and CDC (5%); and Official government sites (4%) (Refer to Figure 1).

### 3.4 Factors associated with eHealth knowledge and practice

Significantly, students from health-related colleges had a higher eHealth literacy score than those in other colleges (53% vs. 13%–15%, *p* = 0.026) (Table 3). Similarly, those who referred to PubMed and Google Scholar for information had higher eHealth literacy than those who did not (47% vs. 30%, *p* = 0.018). Age, gender, university type, and academic performance (measured by grade point average) were not found to be associated with eHealth literacy (*p* > 0.05).

Furthermore, even after adjusting for possible confounders, females were more likely to have higher eHealth literacy than males (OR = 2.03, 95% CI = 1.11–3.78, *p* = 0.023); and those from health-related colleges were more likely to have higher eHealth literacy than those from science and technology colleges (OR = 2.17, 95% CI = 1.04–4.74, *p* = 0.044) (Table 4).

**Table 4:** Univariate and multivariable logistic regression for association between socio-demographic characteristics and high eHealth literacy.

Characteristic	cOR (95% CI)	*p*-value	aOR (95% CI)	*p*-value
Age category				
≤20	1.00		1.00	
>20	1.52 (0.87–2.68)	0.150	1.73 (0.95–3.21)	0.078
Gender				
Male	1.00		1.00	
Female	1.72 (0.99–3.05)	0.059	2.03 (1.11–3.78)	0.023*
College				
Science & Technology	1.00		1.00	
Medicine and Health Sciences	2.57 (1.30–5.38)	**0**.**009**	2.17 (1.04–4.74)	0.044*
Others	1.73 (0.73–4.17)	0.200	1.77 (0.73–4.32)	0.200
Grade point average				
<3.3	1.00		1.00	
≥3.3	0.90 (0.51–1.60)	0.700	–	–
PubMed/scholar as a source of information				
No	1.00		1.00	
Yes	2.05 (1.12–3.74)	**0**.**019**	1.59 (0.81–3.10)	0.200

cOR: Crude odds ratio; aOR: Adjusted odds ratio; CI: Confidence interval. Model fit (Pearson Chi-square): X^2^ = 236.43, df = 230, and *p* = 0.371.

* Statistically signficant.

### 3.5 TAME-ChatGPT scores and their relationship with eHealth literacy

**Figure 2:**
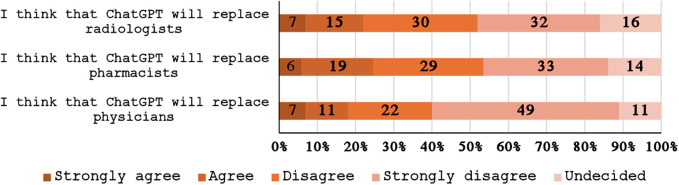
Participants’ opinion about the possibility of ChatGPT replacing health specialists.

Table 4 provides the average TAME-ChatGPT scores for each construct as well as for the individual subscales. The overall TAME-ChatGPT average score for the *usage construct* was 3.2 (SD = 0.41) with *perceived ease of use* subscale (3.87) and *perceived risk of use* subscale (2.45) having the highest and the lowest average scores respectively. The *attitude construct* had an overall score of 2.85 (SD = 0.41) and the *attitude to technology* subscale had the highest average score (3.57) under this construct. The score for *perceived potential to replace healthcare providers* was 2.57 (SD = 0.72) and that of *perceived utility in healthcare* was 2.75 (SD = 0.72). Only the score for *perceived utility in healthcare* was found to be significantly associated with eHealth literacy as those with high e-literacy had higher *perceived utility in healthcare* scores than those with low eHealth literacy (2.93 vs. 2.66, *p* = 0.013). Detailed responses to items assessing the *perceived potential of ChatGPT to replace healthcare providers* are provided in Figure 2 while those for TAME-ChatGPT are provided in Table 5. Table 6 provides the distribution of scores across the three TAME-ChatGPT constructs between participants with low and high eHealth literacy levels.

**Table 5:** Participants’ responses to TAME-ChatGPT.

Characteristic	N (%)
I am concerned about the reliability of the information provided by ChatGPT	
Strongly Agree	49 (21%)
Agree	100 (42%)
Disagree	28 (12%)
Strongly Disagree	10 (4.2%)
Undecided	50 (21%)
I am concerned that using ChatGPT would get me accused of plagiarism	
Strongly Agree	79 (33%)
Agree	92 (39%)
Disagree	29 (12%)
Strongly Disagree	7 (3.0%)
Undecided	30 (13%)
I fear relying too much on ChatGPT and not developing my critical thinking skills	
Strongly Agree	85 (36%)
Agree	74 (31%)
Disagree	32 (14%)
Strongly Disagree	15 (6.4%)
Undecided	30 (13%)
I am concerned about the potential security risks of using ChatGPT	
Strongly Agree	67 (28%)
Agree	82 (35%)
Disagree	39 (16%)
Strongly Disagree	9 (3.8%)
Undecided	40 (17%)
I am concerned about the potential privacy risks that might be associated with using ChatGPT	
Strongly Agree	64 (27%)
Agree	85 (36%)
Disagree	35 (15%)
Strongly Disagree	9 (3.8%)
Undecided	44 (19%)
I am afraid of becoming too dependent on technology like ChatGPT	
Strongly Agree	85 (36%)
Agree	84 (35%)
Disagree	31 (13%)
Strongly Disagree	10 (4.2%)
Undecided	27 (11%)
I am afraid that using ChatGPT would result in a lack of originality in my university assignments and duties	
Strongly Agree	70 (30%)
Agree	97 (41%)
Disagree	27 (11%)
Strongly Disagree	13 (5.5%)
Undecided	28 (12%)
I am afraid that the use of the ChatGPT would be a violation of academic and university policies	
Strongly Agree	70 (30%)
Agree	101 (43%)
Disagree	29 (12%)
Strongly Disagree	7 (3.0%)
Undecided	29 (12%)
I am enthusiastic about using technology such as ChatGPT for learning and research	
Strongly Agree	84 (36%)
Agree	89 (38%)
Disagree	14 (6.0%)
Strongly Disagree	6 (2.6%)
Undecided	42 (18%)
I believe technology such as ChatGPT is an important tool for academic success	
Strongly Agree	69 (29%)
	81 (34%)
Disagree	25 (11%)
Strongly Disagree	10 (4.2%)
Undecided	52 (22%)
I think that technology like ChatGPT is attractive and fun to use	
Strongly Agree	74 (31%)
Agree	113 (48%)
Disagree	15 (6.3%)
Strongly Disagree	7 (3.0%)
Undecided	28 (12%)
I am always keen to learn about new technologies like ChatGPT	
Strongly Agree	80 (34%)
Agree	104 (44%)
Disagree	9 (3.8%)
Strongly Disagree	4 (1.7%)
Undecided	39 (17%)
I trust the opinions of my friends or colleagues about using ChatGPT	
Strongly Agree	37 (16%)
Agree	86 (37%)
Disagree	25 (11%)
Strongly Disagree	9 (3.8%)
Undecided	78 (33%)
ChatGPT helps me to save time when searching for information	
Strongly Agree	82 (35%)
Agree	100 (43%)
Disagree	18 (7.7%)
Strongly Disagree	5 (2.1%)
Undecided	30 (13%)
For me, ChatGPT is a reliable source of accurate information	
Strongly Agree	31 (13%)
Agree	56 (24%)
Disagree	59 (25%)
Strongly Disagree	34 (14%)
Undecided	57 (24%)
ChatGPT helps me in better understanding of difficult topics and concepts	
Strongly Agree	67 (28%)
Agree	98 (41%)
Disagree	20 (8.4%)
Strongly Disagree	6 (2.5%)
Undecided	46 (19%)
I recommend ChatGPT to my colleagues to facilitate their academic duties	
Strongly Agree	52 (22%)
Agree	76 (32%)
Disagree	36 (15%)
Strongly Disagree	16 (6.8%)
Undecided	56 (24%)
I think that using ChatGPT has helped to improve my overall academic performance	
Strongly Agree	46 (19%)
Agree	84 (36%)
Disagree	29 (12%)
Strongly Disagree	15 (6.4%)
Undecided	62 (26%)
I think that using ChatGPT will help me in the diagnosis of my symptoms	
Strongly Agree	23 (9.7%)
Agree	50 (21%)
Disagree	60 (25%)
Strongly Disagree	47 (20%)
Undecided	57 (24%)
I think that using ChatGPT will help me in the diagnosis of the symptoms of my patients	
Strongly Agree	18 (9.0%)
Agree	51 (25%)
Disagree	38 (19%)
Strongly Disagree	56 (28%)
Undecided	38 (19%)
I think that using ChatGPT will help me in understanding the hospital lab reports of myself/my family	
Strongly Agree	23 (9.7%)
Agree	76 (32%)
Disagree	42 (18%)
Strongly Disagree	32 (14%)
Undecided	64 (27%)
I think that using ChatGPT will help me in understanding the lab reports of my patients	
Strongly Agree	36 (18%)
Agree	46 (23%)
Disagree	41 (20%)
Strongly Disagree	32 (16%)
Undecided	48 (24%)
I spontaneously find myself using ChatGPT when I need information for my university assignments and duties	
Strongly Agree	32 (14%)
Agree	96 (41%)
Disagree	47 (20%)
Strongly Disagree	29 (12%)
Undecided	33 (14%)
I appreciate the accuracy and reliability of the information provided by ChatGPT	
Strongly Agree	23 (9.7%)
Agree	69 (29%)
Disagree	52 (22%)
Strongly Disagree	26 (11%)
Undecided	67 (28%)
ChatGPT is easy to use	
Strongly Agree	113 (48%)
Agree	98 (42%)
Disagree	3 (1.3%)
Strongly Disagree	3 (1.3%)
Undecided	19 (8.1%)
The positive experiences of others have encouraged me to use ChatGPT	
Strongly Agree	63 (27%)
Agree	99 (42%)
Disagree	21 (8.9%)
Strongly Disagree	7 (3.0%)
Undecided	47 (20%)
I think using ChatGPT is important for me to keep up with my peers academically	
Strongly Agree	35 (15%)
Agree	65 (27%)
Disagree	61 (26%)
Strongly Disagree	24 (10%)
Undecided	52 (22%)

**Table 6:** Distribution of scores across TAM-ChatGPT constructs and eHealth literacy score.

Domain	Sub-scale	Average TAM-ChatGPT score (Mean ± SD)			
		Overall	eHealth literacy		
			High	Low	*p*-value
Usage construct	Perceived usefulness	3.28 ± 0.54	3.31 ± 0.56	3.27 ± 0.53	0.538
	Behavior/cognitive factors	3.22 ± 0.90	3.15 ± 0.92	3.26 ± 0.89	0.383
	Perceived risk of use	2.45 ± 0.60	2.40 ± 0.55	2.47 ± 0.63	0.358
	Perceived ease of use	3.87 ± 0.41	3.90 ± 0.30	3.85 ± 0.45	0.375
	Construct overall score	3.20 ± 0.41	3.19 ± 0.40	3.21 ± 0.42	0.776
Attitude construct	Perceived risk	2.50 ± 0.57	2.43 ± 0.54	2.54 ± 0.58	0.189
	Anxiety	2.48 ± 0.68	2.45 ± 0.71	2.49 ± 0.67	0.670
	Attitude to technology	3.57 ± 0.46	3.60 ± 0.50	3.56 ± 0.43	0.501
	Construct overall score	2.85 ± 0.41	2.83 ± 0.41	2.86 ± 0.41	0.552
Health care application	Perceived utility in healthcare	2.75 ± 0.79	2.93 ± 0.79	2.66 ± 0.78	0.013*
	Perceived potential to replace healthcare providers	2.57 ± 0.72	2.65 ± 0.80	2.53 ± 0.68	0.232

* Statistically signficant.

## 4 Discussion

This study aimed to assess eHealth literacy levels and associated factors among the university students in the United Arab Emirates. It also aimed to understand attitudes and behaviors of the respective students towards ChatGPT usage. The results reported that mean eHealth literacy score of the participants was 29.3. Female gender, studying in healthcare discipline and the use of specific search engines such as PubMed and Google Scholar was found to be associated with higher eHealth literacy levels. Whereas the average TAME-ChatGPT scores were found to be 3.2 for usage and 2.85 for attitudinal constructs respectively. Additionally, the perceived utility of ChatGPT for healthcare purpose was found to be significantly higher in participants reporting higher eHealth literacy scores.

It was promising to observe higher average score (29.3) on the eHEALS scale among the students in our study. Although no study using the specific scale has been carried out among the university students in the GCC countries, yet recent research surveys conducted among Japanese and Chinese university students reported their eHEALS score being 23.6 (±6.8) and 26.75 (±5.86) respectively (24, 25). The possible reasons could be heavy investment in digital infrastructure by the UAE government resulting in higher internet access and penetration among the population (26). Additionally, the UAE's multicultural environment can also be a contributing factor to foster greater health information-seeking behaviors and cross-cultural health literacy practices (27).

It is noteworthy that the majority of students in our study expressed high confidence in accessing health information i.e., “the utilization of the internet to access health resources on the internet” (84%) and to answer their questions about health (86%). Their level of confidence towards evaluation of health resources in terms of quality was moderate (70%–72%) but was lower (56%) on the application of acquired information to make health related decisions. The findings indicate that students are comfortable in technical aspects of information retrieval but hesitate in its practical application. These results are consistent with findings from a previous study carried out in 2023 in Emirati adolescents and young adults, that assessed their utilization of social media for health information. The survey reported that 74.7% of the study participants were able to obtain the useful health information, with merely 40% having had a health decision influenced by it (17). The reason could be potential difference between actual and perceived health literacy, with students being overtly confident in reporting eHealth literacy and reporting higher levels than actually present. This finding has also been reported previously in a systematic review summarizing eHealth literacy among college students (28). Moreover another possible explanation is the lack of skill set required, as information retrieval is related to technical and search skills, whereas application is based on behaviour change and implementation skills. The difference evident in acquiring knowledge and subsequently translating it to action requires targeted action and guidance for the students such as more hands-on, case-based learning as well as inclusion of decision-making scenarios in the curriculum, in order to overcome the implementation gap.

The difference of gender in the overall digital health literacy scores reflects the variations in health information seeking behavior among different sociodemographic groups. The finding that females tend to have higher eHealth scores than males contrasts to what has been reported among university students in other countries of MENA region such as Iran and Egypt, where research indicates mixed results (14, 29). However, studies carried out in among general public in other MENA countries such as Kuwait and Jordan indicated that the eHEALS scores are less in males compared to females (30, 31). Globally female gender has been found to be significantly associated with online information seeking, resulting in a positive influence on their health (32). Future research would benefit from further studies to explore specific Internet search and retrieval characteristics in relation to gender of students.

Similarly, the findings that the students undertaking studies in healthcare disciplines tend to have higher digital health literacy scores than those studying non health related subjects, corroborate to what has been reported in previous studies (23). One possible explanation for the results could be higher exposure of healthcare concepts among students undertaking degrees in healthcare disciplines, that naturally increases their digital health literacy. Nonetheless, it also urges for enhanced strategies to foster digital health skills in students in non-health related educational fields. This can be achieved by integration of digital health literacy across all educational levels, spanning from elementary, to high school via research projects and university-level critical evaluation courses. Age-appropriate activities can be embedded within existing curricula, supported by teacher training, community partnerships, and performance-based assessments, hence also making use of cross disciplinary opportunities.

Another interesting finding outlined in the results, indicated higher employment of specific search engines such as Google Scholar and PubMed (25%) as compared to general information sources such as Google and Wikipedia being less common (5%). Moreover, participants using PubMed and Google Scholar also reported higher eHealth literacy scores. It implies the importance of understanding of evidence-based information among students, yet there also seems underutilization of official government and specific health organization websites, such as CDC and WHO. These websites offer important avenues in accessing the latest public health guidelines and are crucial to promote such sources for enhanced student awareness. Possible reasons could include less awareness about these credible sources, complex language and technical content along with possible lack of trust. More interaction with these resources can be achieved by enhancing awareness of these platforms among students, improving accessibility, and investing effort towards building trust.

It was observed in our study that among the subscales for usage constructs of TAME-ChatGPT, perceived ease of use scored the highest (3.87), followed by perceived usefulness with perceived risk of use being the lowest (2.45). For the attitude construct, use of technology was the highest scoring subscale whereas anxiety subscale was the one with lowest score (2.48). Higher perception with increased ease of use and usefulness indicates that the tool is considered convenient by the students. Certain notable characteristics of the tool such as friendly user interface, adaptive responses, conversational nature and minimal training requirements make ChatGPT usable and accessible for educational purposes (33). However, the finding that scores on the subscales on risk perception were low, raises concerns on the actual understanding of students about the risks. It warrants more training among students regarding data security, privacy issues, ethical considerations, and responsible use of artificial intelligence (34). Lower scores on perceived potential to replace healthcare professionals such as physicians, radiographers and pharmacists reflect higher trust of students on human healthcare providers, their recognition of the limitations of artificial intelligence and role of clinical expertise in healthcare. A recent observational study in Sweden, that compared the performance of ChatGPT 4 and family medicine specialists indicated that ChatGPT did not perform better than doctors, due to medical inaccuracy (35). The results support the notion that LLMs can potentially be considered as complimentary and augmentative tool to help healthcare professionals rather than complete replacement.

This study also explored the relationship between levels of eHealth literacy and attitudes towards ChatGPT usage. Interestingly students with higher eHealth scores indicated higher scores for subscale on perceived use of ChatGPT in healthcare. This implies that students equipped with better digital health literacy skills may leverage the AI tools in a better way. However, it is important to note that students need to be well versed in guidelines for appropriate AI use in healthcare education is important as in the critical evaluation of the content generated by AI.

This study, to our knowledge was the first one to assess digital health literacy levels using eHEALS tool, among the students undertaking studies in the universities of UAE. We also assessed the relationship between the literacy levels and attitudes towards ChatGPT usage. However, the results need to be interpreted with caution as the responses were self-reported and may be subjective to social desirability bias that may lead to over reporting of preferred behaviors or recall bias resulting in inaccurate estimates. This could be addressed by combining and triangulating self-reported measures with objective assessments of eHealth literacy. Another key limitation of this study is the low response rate, with only 237 complete responses obtained from an estimated national student population of approximately 100,000 across both public and private universities. Although we reached out to all licensed universities in the UAE through formal emails to their administrative offices and promoted participation through academic events such as undergraduate and postgraduate research conferences, response remained limited. The use of digital platforms for survey dissemination, supplemented by snowball sampling, may have introduced non-response bias by overrepresenting students who are more digitally engaged, academically active, or better connected within social or institutional networks. Additionally, the under-representation of students from private universities (25%) relative to the national mix, was also evident. This potentially limits the generalizability of the findings to the broader student population, particularly those who may have lower digital literacy, less interest in health-related technologies or from private institutions. Additionally, the study followed a cross-sectional design, preventing exploring causal relationships, that can be replaced by longitudinal study designs to track response consistency over time. Nevertheless, this study provides an insight of digital health literacy and acceptance of an AI based tool such as ChatGPT, revealing moderate to high overall eHEALS score. Findings indicate proficient information to navigate health information, but the increasing digital landscape urges our students to be even more competent, focusing on the room for improvement. Moreover, the lack of confidence in application of the acquired information warrants actionable steps such as curriculum development that bridges the gap between technical skills and practical application. Education interventions are also required to address the disparity in eHealth literacy among students from varied academic disciplines.

Future research exploring barriers and facilitators towards decision making processes in the use of AI based models such as Chat GPT can also be employed, offering a deeper understanding of the challenges and opportunities in this area. Future studies could explore longitudinal data to examine how eHealth literacy and AI usage evolve over time.

The results underscore the importance of development of educational approaches that enhance digital health literacy as well as AI competency among the students so that they are better equipped for the upcoming digital era. These approaches can include mandatory courses or short digital health modules embedded into existing curriculum. Moreover, partnerships with healthcare organizations and tech companies, offering practical experience to the students as well as lectures and workshops by experts and guest speakers can be beneficial.

## Data Availability

The original contributions presented in the study are included in the article/Supplementary Material, further inquiries can be directed to the corresponding author.
